# State of the art: Proceedings of the American Association for Thoracic Surgery Enhanced Recovery After Cardiac Surgery Summit

**DOI:** 10.1016/j.xjon.2023.04.004

**Published:** 2023-04-15

**Authors:** Subhasis Chatterjee, Rakesh C. Arora, Cheryl Crisafi, Shannon Crotwell, Marc W. Gerdisch, Nevin M. Katz, Kevin W. Lobdell, Vicki Morton-Bailey, John P. Pirris, V. Seenu Reddy, Rawn Salenger, Dirk Varelmann, Daniel T. Engelman

**Affiliations:** a Baylor College of Medicine & Thoracic Surgery ICU/ECMO, Texas Heart Institute, Baylor St Lukes Medical Center, Houston, Tex; b Perioperative and Cardiac Critical Care, Harrington Heart Vascular Institute at University Hospitals, Cleveland, Ohio; c Cardiac Surgery, Baystate Medical Center, Springfield, Mass; d Cardiac Surgery Program Development, Sanger Heart & Vascular Institute, Atrium Health, Charlotte, NC; e Cardiothoracic Surgery, Franciscan Health, Indianapolis, Ind; f Division of Cardiac Surgery, Department of Surgery, Johns Hopkins University School of Medicine, Baltimore, Md; g Cardiovascular Quality, Education and Research, Sanger Heart & Vascular Institute, Charlotte, NC; h Clinical and Quality Outcomes, Providence Anesthesiology Associates, Charlotte, NC; i Cardiothoracic Surgery, University of Florida Health, Jacksonville, Fla; j Cardiac Surgery, ERAS Program, TriStar Centennial Medical Center, Nashville, Tenn; k Department of Surgery, University of Maryland School of Medicine, Baltimore, Md; l Cardiac Surgery Intensive Care Unit, Department of Anesthesiology, Perioperative and Pain Medicine, Brigham and Women's Hospital, Harvard Medical School, Boston, Mass; m Department of Surgery, Baystate Medical Center, University of Massachusetts-Baystate, Springfield, Mass

**Keywords:** cardiac surgery, enhanced recovery, perioperative care

## Abstract

Despite the benefits established for multiple surgical specialties, enhanced recovery after surgery has been underused in cardiac surgery. A cardiac enhanced recovery after surgery summit was convened at the 102nd American Association for Thoracic Surgery annual meeting in May 2022 for experts to convey key enhanced recovery after surgery concepts, best practices, and applicable results for cardiac surgery. Topics included implementation of enhanced recovery after surgery, prehabilitation and nutrition, rigid sternal fixation, goal-directed therapy, and multimodal pain management.

**Figure:**
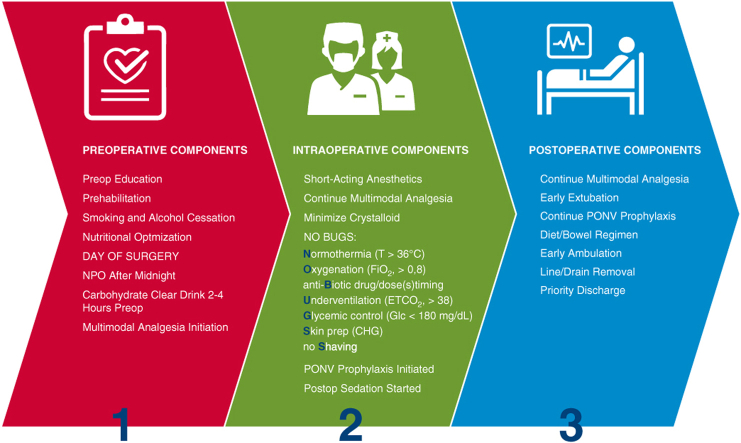
Potential perioperative care bundles for enhanced recovery after CS.

**Central Message**

Implementation of ERAS Cardiac Consensus Guidelines can reduce complications and length of stay and expedite patient recovery. Multidisciplinary teams and hospital administration support are essential.

**Perspective**

Enhanced recovery after CS provides an evidence-based, patient-centric approach to perioperative care to optimize recovery throughout a patient's surgical journey. From the 102nd Annual American Association for Thoracic Surgery meeting, the ERAS Cardiac Summit talks are summarized. The topics ranged from program building, preoperative optimization, and GDT to prevention of postoperative complications.

Enhanced recovery after surgery (ERAS) initiatives embrace multidisciplinary implementation of patient-focused, evidence-based protocols for all phases of the patient surgical journey. The goal is to minimize patient emotional and physiologic stress, reduce postoperative complications, and expedite a patient's return to normal activities. ERAS is commonly used for a host of surgeries, including colorectal, foregut, and gynecological, among others; however, adoption in cardiac surgery (CS) has been a more recent development. At the 102nd meeting of the American Association for Thoracic Surgery in Boston, Massachusetts in May 2022, a summit was convened of ERAS experts to discuss application of ERAS concepts to CS, with each topic covering introductory and advanced curricula. The intent of this endeavor is to provide a summary from the transcript that was taken in real-time during the summit. The goal is to provide a reader an overview of the presentations during the session with accompanying references to support statements made during this session. Individual speakers may provide single-center results; these should be interpreted in the clinical context. For additional details, the reader is encouraged to read published ERAS Cardiac guidelines and selected references for more detail on any aspect of ERAS.^1^

## Starting an Enhanced Recovery After Surgery Program

The components of ERAS—implementation of evidence-based protocols, adoption of patient-centered care, reduction in unnecessary variations, and improvement in quality, safety, patient and provider satisfaction—often necessitates a change in departmental culture. This begins with team building, according to presenters Cheryl Crisafi, MSN, RN, CNL, Cardiac Surgery Program Coordinator Nurse at Baystate Medical Center, and Shannon Crotwell, BSN, RN, CCRN, Cardiac Surgery Program Development at Atrium Health. The CS ERAS team should include surgeons, anesthesiologists, critical-care practitioners, nurses, advanced practice providers, a program coordinator, and institutional leadership. Representatives from other disciplines, such as pharmacists, respiratory therapists, dietitians, physical therapists, data analysts, and case managers, are also integral members of the broader team. Nascent CS ERAS initiatives need champions to advance the project and help overcome challenges; these may stem from failures to break down silos between phases of surgical care, to prepare for staff turnover and the need for ongoing education, or to generate buy-in for ERAS among critical stakeholders. Although core ERAS components have been identified, each local institution needs to tailor its ERAS program to their local staffing, surgical volume, and other considerations. There is not a “one-size-fits-all” approach (Table 1).

**Table 1:** Recommended aspects of a cardiac enhanced recovery after surgery program

Phase	Key components
Starting an ERAS program	Build multidisciplinary team
	Identify program “champions”
	Get buy-in from hospital administrators
	Choose preoperative, perioperative, and postoperative care bundles
	Tailor to staffing, surgical volume, and other facility considerations
	Create dashboards, integrate into electronic medical records
Preoperative	Consider prehabilitation (anemia, nutrition, frailty, glucose)
	Perform a kidney health assessment
	Continue food intake until 8 h before anesthesia
	Include oral carbohydrate loading before surgery in noninsulin-dependent diabetic patients
	Continue liquid intake until 2-4 h before anesthesia
Intraoperative	Implement multimodal opioid-sparing pain management
	Apply evidence-based strategies to reduce surgical site infections
	Consider rigid sternum fixation
	Institute GDT
Postoperative	Implement KDIGO care bundle in appropriate patients
	Use multimodal opioid-sparing pain management
	Institute GDT
	Resume nutrition early
	Avoid hypothermia
	Remove tubes and drains early

*ERAS*, Enhanced recovery after surgery; *GDT*, goal-directed therapy; *KDIGO*, Kidney Disease Improving Global Outcomes.

**Figure 1:**
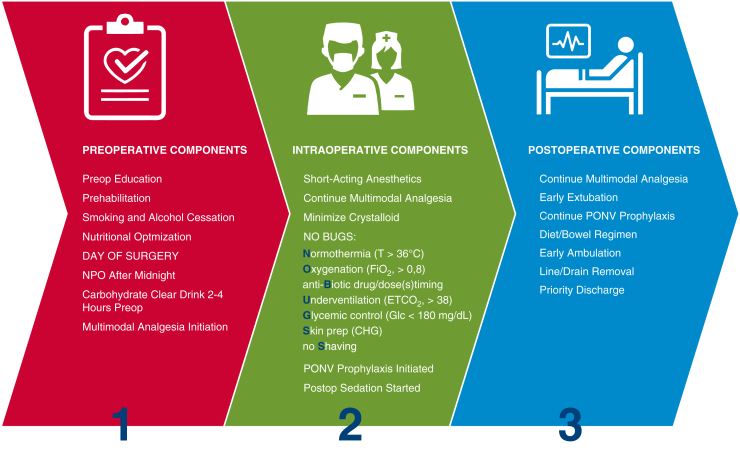
Potential cardiac ERAS care bundles. When implementing a new CS ERAS program, the number and variety of potential care bundles can be overwhelming. Programs may benefit from starting with a manageable number of care bundles and building on success. *NPO*, Nothing by mouth; *ETCO2*, end-tidal carbon dioxide; *CHG*, Chlorhexidine gluconate; *PONV*, postoperative nausea and vomiting.

**Figure 2:**
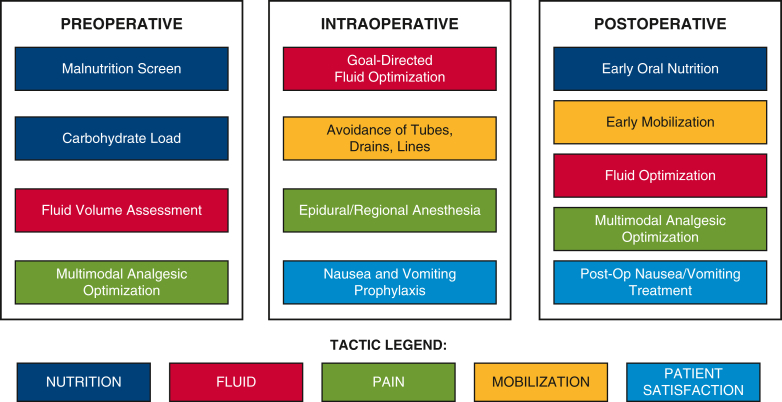
Sample care bundles and surgical phase tasks. To implement a new cardiac ERAS program at TriStar Centennial Medical Center, new protocols were created by applying selected cardiac ERAS care bundles across perioperative phases.

V. Seenu Reddy, MD, MBA, Chief, Cardiac Surgery and Director, ERAS Program at TriStar Centennial Medical Center, provided insight into partnering with hospital administrators who often are focused on the costs of complications and variations in processes of care. ERAS pathways can reduce variability, benefiting a hospital's bottom line through reduction in hospital stays and costs. However, with approximately 20 potential care bundles, CS ERAS protocol implementation can be overwhelming at the outset (Figure 1). Dr Reddy's facility opted to focus on 6 areas: patient education, preoperative hydration, multimodal pain management, goal-directed fluid management, early postoperative mobilization and intake of food and liquids, and a multidisciplinary approach to implementation and adoption of protocols. These care bundles were then applied to the surgical phases to create protocols for care management (Figure 2). The ability to scale ERAS from one surgical area to others can be enticing for hospital administrators, particularly as ERAS programs contribute to operating margins; shorter length of stay and increased bed turnover can offset the upfront investment.^2^

Key Takeaway: Dr Reddy also counseled that administrators are skilled at creating metrics dashboards that marshal resources for web-based materials, including integration of ERAS protocols into electronic medical record systems.

## Prehabilitation and Nutrition

A sizeable portion of patients undergoing CS are frail, a condition associated with a 5-fold increase in the risk for major adverse cardiovascular and cerebrovascular events.^3^ More than 10% of patients undergoing CS are readmitted within 30 days and approximately 20% of are readmitted within 65 days.^4^^,^^5^

Rakesh C. Arora, MD, PhD, Director of Perioperative and Cardiac Critical Care at University Hospitals, Cleveland, Ohio, encouraged participants to assess frailty by using a tool that is easily implementable within the clinical context. One such tool is www.frailtytool.com, which can be easily implemented in the outpatient clinic to help determine a patient's frailty and start the conversation with the interdisciplinary team on whether the patient would benefit from prehabilitation to augment functional capacity. Dr Arora noted several randomized trials are evaluating the effect of exercise prehabilitation in CS patients with frailty: Patients have demonstrated exercise tolerance with improved performance on the 6-minute walk test and gait speed. In abdominal surgeries, prehabilitation has augmented postoperative walking capacity and, in one study, lowered complications.^6^, ^7^, ^8^ Thus, Dr Arora posed a question for attendees to consider: “In some patients, is it better to delay surgery to optimize functional capacity before surgery?” Upcoming randomized trials may offer insight into that question.

**Figure 3:**
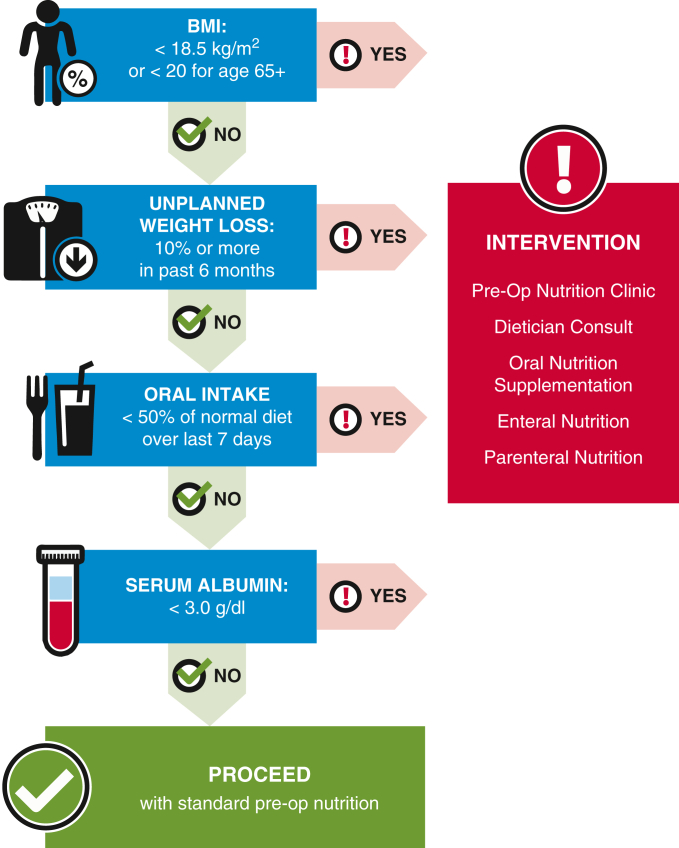
Perioperative nutrition score tool. A tool for identifying patients in need of perioperative nutritional intervention, based on a joint consensus report from the American Society for Enhanced Recovery and the Perioperative Quality Initiative.

Adapted from Wischmeyer and colleagues.^12^*BMI*, Body mass index.

Approximately 20% of CS patients are malnourished,^9^ which can lead to worse functional outcomes, higher rates of infection and pneumonia, and increased hospital and intensive care unit (ICU) stays.^10^ Vicki Morton-Bailey, DNP, AGNP-BC, Director, Clinical and Quality Outcomes, Providence Anesthesiology Associates, provided an overview of relevant CS ERAS recommendations: complete a detailed nutritional history, assess body composition, and include nutrition intervention in the overall care plan. The Perioperative Nutrition Screen tool was developed to identify patients with a nutrition imbalance (Figure 3). Mildly malnourished individuals should begin nutrition optimization 7 to 10 days before surgery. Protein intake, which should be prioritized over a total caloric goal, should exceed 1.2 g/kg/d for individuals with nutritional risk; immunonutrition with arginine or fish oil also can be considered. These targeted interventions will need randomized trials to determine their efficacy by demonstrating a reduction in adverse events. Meanwhile, before surgery, solid foods can be consumed up to 8 hours before surgery, and oral carbohydrate loading may be given before surgery. Consumption of clear liquids should continue until 2 to 4 hours before anesthesia.^11^^,^^12^ Underfeeding of CS patients is common, with postoperative calorie and protein intake often being inadequate.^13^ Nutrition should be given early postoperatively. Oral nutrition is preferred to enteral, and enteral nutrition is preferred to parenteral, although the combination of enteral and parenteral nutrition should be considered in patients unable to meet full nutrition goals with enteral alone.^12^

Key Takeaway: Although there is not yet a “gold standard” for measuring frailty in the preoperative setting, the contemporary perioperative team should perform an assessment to determine vulnerability in patients undergoing CS and seek opportunities to optimize identified risk, if feasible, before surgery.

## Rigid Sternal Fixation

Wire cerclage, which is commonly used to close the sternum using compressive force after median sternotomy, does not prevent postoperative sternal movement.^1^ Complications occur in 0.5% to 5.0% of patients, with 0.2% to 3.0% of individuals developing mediastinitis.^14^ John Pirris, MD, Chief of Cardiothoracic Surgery, University of Florida Health-Jacksonville, achieves rigid sternal fixation with titanium plates and titanium screws. Rigid plate fixation improves stabilization of sternal edges, which expedites healing and can foster a reduction in postoperative complications and opioid use, as well as hasten recovery.^15^, ^16^, ^17^ In the rare event a patient needs to be reopened on an emergency basis, the titanium plates are designed to be cut with standard wire cutters, addressing a common concern. Dr Pirris postulated that pain reduction is perhaps the greatest benefit of rigid plate fixation, which has implications for total overall costs. At his facility, hospital readmissions have decreased by 23% since implementation of rigid plate fixation.

Marc W. Gerdisch, MD, Chief of Cardiothoracic Surgery at Franciscan Health, advocated for an aggressive process to reverse physical trauma and eliminate pain. For patients undergoing sternotomy, rigid plate fixation is part of his approach. According to Dr Gerdisch, complete sternal stabilization achieves greater patient comfort and range of movement. Indeed, the bone will heal more rapidly. More important, the patient is immediately able to use their upper body in a normal manner to get out of bed, sit and rise from a chair or toilet, or bear weight with the aid of a walker. Greater mobility is accompanied by minimal pain medication, improved bowel function, and less delirium.^18^ After rigid plate fixation was adopted at Dr Gerdisch's facility, narcotic use after sternotomy decreased by 87% in morphine milligram equivalents from 2015 to 2020. Approximately half of patients did not use any narcotics postoperatively in 2020. Minimal pain and full mobility also translated to fewer patients being discharged to a skilled nursing facility (10% of patients in 2020 vs 30% in 2015). This has potential benefits in terms of reimbursement, which continues to transition to value-based care.^19^ Rigid plate fixation has a higher upfront cost, but by improving patient comfort, decreasing pain, reducing narcotic use, increasing mobility, decreasing readmissions, and minimizing discharge to skilled nursing, it can lower overall costs. These observations have been supported by a recent meta-analysis of 3 randomized trials (N = 427) and 5 observational studies (N = 1025) with the major benefits in sternal complications seen in high-risk patients and the major cost benefits seen in observational trials. Additional randomized trials are needed to better define the benefits.^20^

Key Takeaway: Observations of the potential benefit of rigid sternal fixation have been supported by a recent meta-analysis.^20^ Major benefits in sternal complications are seen in high-risk patients, and the major cost benefits are seen in observational trials. Additional randomized trials are needed to better define the benefits.

## Surgical Site Infection

Rawn Salenger, MD, Associate Professor of Surgery, University of Maryland School of Medicine, provided an overview of the ERAS Cardiac care bundle of evidence-based strategies to reduce CS-related infections, which has a class I recommendation in the ERAS Cardiac Society guidelines.^1^ The care bundle should include preoperative topical nasal decolonization and intravenous (IV) administration of cephalosporin 30 to 60 minutes before surgery (Table 2).^1^ It should also take into account the center's antibiogram and local antimicrobial resistance patterns. Dr Salenger commented that cephalosporin should be given to patients with a penicillin allergy. The care bundle may also include clipping instead of shaving and application of chlorhexidine-alcohol-based solution on the skin before surgery. The most recent CS ERAS guidelines, issued in 2019, indicate that the operative wound dressing should be removed after 48 hours.^1^ However, Dr Salenger noted that this may be revisited in updated guidelines, given the effectiveness of primary negative pressure wound therapy and different needs related to silver-impregnated dressings. Dr Salenger also encouraged attendees to consider data elements to be collected in CS ERAS programs to track outcomes and assess compliance with ERAS protocols.^21^

**Table 2:** Surgical site infection bundle recommended for cardiac enhanced recovery after surgery

Level of evidence by classification of recommendation	Recommendation
Class I	
A	Perform topical intranasal decolonization before surgery
A	Administer IV cephalosporin prophylactic antibiotic 30-60 min before surgery
C	Clipping (vs shaving) immediately before surgery
Class IIb	
C	Use a chlorhexidine alcohol–based solution for skin preparation before surgery
Class IIa	
C	Remove operative wound dressing after 48 h

Source: Engelman and colleagues.^1^*IV*, Intravenous.

Key Takeaway: Knowing a center's antibiogram and local antimicrobial resistance patterns along with tracking center-level data is important.

## Goal-Directed Therapy

Dr Reddy presented on hemodynamic management, which often varies between clinicians and facilities, as it typically reflects standards learned by the cardiac surgeon during residency/fellowship training. Dr Reddy cautioned that volume management based on a patient's weight and/or the expected surgical trauma disregards other needs, such as oxygen delivery.^22^ In addition, management goals shift during the perioperative phases of surgery when the patient may experience rapid hemodynamic alterations. Without physiology-focused fluid management, there is risk of hypovolemia, which can lead to inadequate postoperative oxygen delivery and tissue perfusion,^23^ or fluid overload, which can also increase complications, hospital stays, and mortality rates.^24^ Of note, 85% of current fluid bolus decisions in the ICU are made by nurses or junior house-staff, thus providing a rational approach to resuscitation is necessary.^25^

Goal-directed therapy (GDT) is a key component of ERAS programs to reduce variability and provide continuous advanced monitoring for physiology-driven administration of fluids and pharmacotherapies. GDT has been shown to reduce morbidity in CS^26^^,^^27^ as well as hospital length of stay.^26^, ^27^, ^28^ GDT can reduce the incidence and severity of cardiac surgery–associated acute kidney injury (CSA-AKI) through the use of a Kidney Disease Improving Global Outcomes (KDIGO) bundle of care,^29^ which lowers overall healthcare costs.^30^

The overall goal is adequate tissue perfusion. Advanced monitoring systems detect hemodynamic instability, and aid in differentiation of the potential causes, to facilitate titration of the appropriate therapy to restore the balance of oxygen delivery and consumption. According to Dr Reddy, the physiologic reason to perform a fluid change is when the stroke volume (SV) is low. However, patients who have little stroke volume variation (SVV) are not responsive to fluids. Therefore, only those who are responsive to fluids should be resuscitated with fluid boluses; for others, alternative strategies are necessary.

In the advanced hemodynamic monitoring session, Subhasis Chatterjee, MD, Thoracic Surgery, ICU, and ECMO Program Director, Baylor St Lukes Medical Center, noted that since ICU staffing coverage models differ greatly across institutions with a variety of healthcare providers managing patients, standardizing care algorithms has intuitive appeal. Although GDT technology does not replace clinical decision making, it guides the entire clinical team to maintain a consistent approach to achieving the optimal balance of fluids, inotropes, and vasopressors. GDT algorithms were first developed to optimize SV. Dr Chatterjee commented that algorithms attuned to SVV may be more preferable in CS although they may be limited in patients with severe ventricular dysfunction and have not been validated in patients with arrhythmias.

Numerous studies have demonstrated the benefit of GDT with algorithms focusing on SVV, cardiac index, systemic vascular resistance, or mean arterial pressure. These GDT algorithms have demonstrated a reduction in postoperative pulmonary complications,^31^ significantly reduced the incidence of acute kidney injury (AKI) within 72 hours postoperatively,^29^ lowered overall complications, and enabled faster weaning of vasoactive medications and inotropes.^27^ Dynamic arterial elastance is a more sophisticated parameter in some GDT technologies. Dynamic arterial elastance, the ratio of pressure pulse variation to SVV, measures afterload with a high degree of reliability.^32^

Key Takeaway: GDT is a useful adjunct in patient assessment to aid in clinical decision making.

## Preservation of Kidney Health

CSA-AKI occurs in 20% to 40% of cases,^33^ and more than 25% of patients with CSA-AKI are rehospitalized within 30 days of the index procedure, as even mild kidney injury can lead to readmission.^34^ CSA-AKI increases the odds of mortality by more than 4-fold.^35^ Lack of blood flow to the kidneys can cause acute kidney stress and lead to CSA-AKI. Nevin Katz, MD, Professor Emeritus, John Hopkins University, provided an overview of risk factors for CSA-AKI, including acuity and complexity of surgeries, age, gender, preoperative renal disease, low ejection fraction, diabetes, use of an intra-aortic balloon pump, emergency surgery, mitral valve surgery, and reoperation. In addition, cardiopulmonary bypass risk factors, such as duration of use, arterial cannulation site, and antegrade versus retrograde perfusion, can lead to CSA-AKI. Urine output can be a surrogate for kidney perfusion,^36^ but Dr Katz noted that current methods for urine output monitoring are outdated and cumbersome.

Daniel Engelman, MD, Professor of Surgery, University of Massachusetts-Baystate, Baystate Medical Center, expanded on CSA-AKI, with an overview of guideline-recommended GDT to reduce postoperative complications. The ERAS Cardiac guidelines also endorse use of the KDIGO bundle of care: avoid use of nephrotoxic agents, discontinue angiotensin-converting enzyme inhibitors and angiotensin receptor blockers for the first 48 hours after surgery, monitor serum creatinine levels and urine output, avoid hyperglycemia in the first 72 hours after surgery, use alternatives to radiocontrast agents, and closely monitor hemodynamics using a prespecified algorithm.^1^ Biomarkers of cellular stress may identify patients at risk for AKI. One study found that cellular stress biomarkers, tissue inhibitor of metalloproteinase 2 and insulin-like growth factor-binding protein 7, predicted onset of moderate and severe CSA-AKI.^37^ Dr Engelman explained how use of an Acute Kidney Response Team and a stepped alarm system starting with urinary biomarker testing on postoperative day (POD) 1 directs care at his facility. Patients with no or negligible risk of AKI are fast-tracked for transfer out of the ICU, those with low positive levels of biomarker are monitored and transferred to telemetry later in the day, and patients with high biomarker levels activate the Acute Kidney Response Team for aggressive goal-directed fluid therapy and the KDIGO bundle. The number of patients who have postoperative stage 2 or 3 AKI has been reduced by 84.9% at Dr Engelman's facility using this approach.^38^ Notably, this biomarker guided algorithm is not suitable for patients with preoperative serum creatinine >2 mg/dL, those on dialysis, and those who received methylene blue.

Key Takeaway: AKI is common and associated with increased mortality. The use of an Acute Kidney Response Team may be effective in reducing the occurrence or progression of AKI after CS.

## Pain Management

Multimodal therapy uses multiple nonopioid agents or techniques, such as a regional block, to reduce the amount of opioids used in postoperative pain management. Dr Salenger presented the protocol used at his facility for CS patients: ketamine 0.5 mg/kg IV bolus started in the operating room, with a drip in the ICU; dexmedetomidine IV started before leaving the operating roomn and continued in the ICU; gabapentin 300 mg by mouth every 8 hours through POD 4 or 100 mg for patients aged more than 75 years; and acetaminophen 1 g by mouth every 6 hours through POD 4. Since implementation of this care bundle in 2019, the use of fentanyl in the first 24 postoperative hours decreased to 93.3 μg from 272.8 mL before ERAS implementation (*P* < .01), and the amount of morphine milligram equivalents declined to 55.8 mg from 87.6 mg (*P* < .01). Patients under the ERAS pathway received fewer morphine milligram equivalents through POD 4 compared with those treated before ERAS implementation. Patients using the ERAS multimodal pathway had greater mobility, less postoperative nausea, less lightheadedness, and a shorter postoperative length of stay compared with those without ERAS multimodal management.

Dirk Varelmann, MD, Medical Co-Director, Cardiac Surgery Intensive Care Unit, Department of Anesthesiology, Perioperative and Pain Medicine, Brigham and Women's Hospital, Harvard Medical School, encouraged clinicians to be proactive with multimodal analgesia, with around-the-clock dosing postoperatively and using opioids as a supplement for nonopioid treatments. Dr Varelmann recommended a standing order for acetaminophen 650 to 1000 mg every 6 hours postoperatively. Ketorolac, a nonsteroidal anti-inflammatory drug, is contraindicated in patients with coronary artery disease and has a black box warning for bleeding and cardiovascular thrombosis. However, if clinicians choose to use ketorolac, Dr Varelmann advised dosing at 15 mg, not 30 mg, because the higher dose does not provide greater pain management and has increased side effects. Nonselective cyclooxygenase inhibitors, such as ibuprofen, are preferred to selective cyclooxygenase inhibitors. Nonsteroidal anti-inflammatory drugs should be used with caution in patients with thrombosis, bleeding, and renal insufficiency. Early studies of gabapentinoids showed that a single preoperative dose reduced pain scores and decreased opioid use. However, when used in conjunction with opioids, gabapentinoids can aggravate respiratory depression and may increase ventilator duration. Dosing of gabapentinoids should be adjusted for age and kidney function. Dexmedetomidine, a selective α_2_ agonist, may reduce time to extubation and decrease the incidence of delirium. It is associated with shorter ICU stays and a reduction in opioid use.

Regional anesthesia, including skin infiltration, parasternal block, and pectoral blocks, can dramatically reduce opioid use. However, regional anesthesia does not address the entire distribution of pain and can be time consuming to implement.

Key Takeaway: Measure opioid use in patients and promote mobilization through effective multimodal (ie, opioid-sparing) analgesia strategies.

## Conclusions

Team building, support from hospital administration, and multidisciplinary adoption are crucial to successful implementation of cardiac ERAS programs. Attention should be paid to which care bundles are included in ERAS pathways. Active research into implementation strategies that increase the dissemination of best practices is vital. Prehabilitation with exercise and nutrition, rigid sternal fixation, GDT, and multimodal pain management can reduce postoperative complications, shorten hospital and ICU stays, and expedite patient recovery.

### Conflict of Interest Statement

S.C. has served on advisory boards for Edwards Lifesciences, La Jolla Pharmaceutical Company, Baxter Pharmaceuticals, and Eagle Pharmaceuticals. R.C.A. has served on advisory boards for Edwards Lifesciences, HLS Therapeutics, and Abbott Nutrition and has received speaker honoraria for work unrelated to this manuscript. M.W.G. has served as a consultant for Edwards Lifesciences; has served as a consultant, received research support, and/or served on steering committees for Atrivion, CorMatrix, and Zimmer Biomet; has served as a consultant, received research support, and served as a national principal investigator for Atricure and CorMatrix; and has served on advisory boards for PleuraFlow along with research support from DASI Imaging. K.W.L. has served as consultant to Abiomed, Terumo, Medela, and Medtronic. V.M.-B. has served as a consultant for Edwards Lifesciences. J.P.P. has served as a consultant to Zimmer Biomet. V.S.R. has served as a consultant for Edwards Lifesciences. R.S. has served as a consultant to Terumo and on advisory boards for Zimmer and La Jolla Pharmaceutical Company. D.V. has received consulting fees from Encare AB. D.T.E. discloses relationships with Edwards Lifesciences, Rockwell Medical, Astellas Pharma, Alexion, Terumo, Medela, Guard Therapeutics, and Renibus Therapeutics. All other authors reported no conflicts of interest.

The *Journal* policy requires editors and reviewers to disclose conflicts of interest and to decline handling or reviewing manuscripts for which they may have a conflict of interest. The editors and reviewers of this article have no conflicts of interest.

## References

[1] Engelman D.T., Ben Ali W., Williams J.B., Perrault L.P., Reddy V.S., Arora R.C. (2019). Guidelines for perioperative care in cardiac surgery: enhanced recovery after surgery Society Recommendations. JAMA Surg.

[2] Dong Y., Zhang Y., Jin C. (2021). Comprehensive economic evaluation of enhanced recovery after surgery in hepatectomy. Int J Equity Health.

[3] Sepehri A., Beggs T., Hassan A., Rigatto C., Shaw-Daigle C., Tangri N. (2014). The impact of frailty on outcomes after cardiac surgery: a systematic review. J Thorac Cardiovasc Surg.

[4] Iribarne A., Chang H., Alexander J.H., Gillinov A.M., Moquete E., Puskas J.D. (2014). Readmissions after cardiac surgery: experience of the National Institutes of Health/Canadian Institutes of Health research cardiothoracic surgical trials network. Ann Thorac Surg.

[5] Shah R.M., Zhang Q., Chatterjee S., Cheema F., Loor G., Lemaire S.A. (2019). Incidence, cost, and risk factors for readmission after coronary artery bypass grafting. Ann Thorac Surg.

[6] Barberan-Garcia A., Ubré M., Roca J., Lacy A.M., Burgos F., Risco R. (2018). Personalised prehabilitation in high-risk patients undergoing elective major abdominal surgery: a randomized blinded controlled trial. Ann Surg.

[7] Gillis C., Li C., Lee L., Awasthi R., Augustin B., Gamsa A. (2014). Prehabilitation versus rehabilitation: a randomized control trial in patients undergoing colorectal resection for cancer. Anesthesiology.

[8] Li C., Carli F., Lee L., Charlebois P., Stein B., Liberman A.S. (2013). Impact of a trimodal prehabilitation program on functional recovery after colorectal cancer surgery: a pilot study. Surg Endosc.

[9] Hill A., Goetzenich A., Stoppe C. (2022). Commentary: nutritional status before cardiac surgery-at the 11th hour. J Thorac Cardiovasc Surg.

[10] Hill A., Nesterova E., Lomivorotov V., Efremov S., Goetzenich A., Benstoem C. (2018). Current evidence about nutrition support in cardiac surgery patients-What do we know. Nutrients.

[11] Weimann A., Braga M., Carli F., Higashiguchi T., Hübner M., Klek S. (2021). ESPEN practical guideline: clinical nutrition in surgery. Clin Nutr.

[12] Wischmeyer P.E., Carli F., Evans D.C., Guilbert S., Kozar R., Pryor A. (2018). American Society for Enhanced Recovery and Perioperative Quality Initiative joint consensus statement on nutrition screening and therapy within a surgical enhanced recovery pathway. Anesth Analg.

[13] Stoppe C., Goetzenich A., Whitman G., Ohkuma R., Brown T., Hatzakorzian R. (2017). Role of nutrition support in adult cardiac surgery: a consensus statement from an international multidisciplinary expert group on nutrition in cardiac surgery. Crit Care.

[14] Olbrecht V.A., Barreiro C.J., Bonde P.N., Williams J.A., Baumgartner W.A., Gott V.L. (2006). Clinical outcomes of noninfectious sternal dehiscence after median sternotomy. Ann Thorac Surg.

[15] Allen K.B., Thourani V.H., Naka Y., Grubb K.J., Grehan J., Patel N. (2017). Randomized, multicenter trial comparing sternotomy closure with rigid plate fixation to wire cerclage. J Thorac Cardiovasc Surg.

[16] Raman J., Lehmann S., Zehr K., De Guzman B.J., Aklog L., Garrett H.E. (2012). Sternal closure with rigid plate fixation versus wire closure: a randomized controlled multicenter trial. Ann Thorac Surg.

[17] Royse A.G., El-Ansary D., Hoang W., Lui E., McCusker M., Tivendale L. (2020). A randomized trial comparing the effects of sternal band and plate fixation of the sternum with that of figure-of-8 wires on sternal edge motion and quality of recovery after cardiac surgery. Interact Cardiovasc Thorac Surg.

[18] Gerdisch M.W., Allen K.B., Naka Y., Bonnell M.R., Landolfo K.P., Grehan J. (2020). Orthopedic principles to facilitate enhanced recovery after cardiac surgery. Crit Care Clin.

[19] Horvath K.A. (2022). Can cardiothoracic surgeons succeed in value-based care. Ann Thorac Surg.

[20] Tam D.Y., Nedadur R., Yu M., Yanagawa B., Fremes S.E., Friedrich J.O. (2018). Rigid plate fixation versus wire cerclage for sternotomy after cardiac surgery: a meta-analysis. Ann Thorac Surg.

[21] Hirji S.A., Salenger R., Boyle E.M., Williams J., Reddy V.S., Grant M.C. (2021). Expert consensus of data elements for collection for enhanced recovery after cardiac surgery. World J Surg.

[22] Navarro L.H., Bloomstone J.A., Auler J.O., Cannesson M., Rocca G.D., Gan T.J. (2015). Perioperative fluid therapy: a statement from the international Fluid Optimization Group. Perioper Med (Lond).

[23] Aneman A., Brechot N., Brodie D., Colreavy F., Fraser J., Gomersall C. (2018). Advances in critical care management of patients undergoing cardiac surgery. Intensive Care Med.

[24] Stein A., de Souza L.V., Belettini C.R., Menegazzo W.R., Viégas J.R., Costa Pereira E.M. (2012). Fluid overload and changes in serum creatinine after cardiac surgery: predictors of mortality and longer intensive care stay. A prospective cohort study. Crit Care.

[25] Parke R.L., McGuinness S.P., Gilder E., McCarthy L.W. (2014). Intravenous fluid use after cardiac surgery: a multicentre, prospective, observational study. Crit Care Resusc.

[26] Aya H.D., Cecconi M., Hamilton M., Rhodes A. (2013). Goal-directed therapy in cardiac surgery: a systematic review and meta-analysis. Br J Anaesth.

[27] Osawa E.A., Rhodes A., Landoni G., Galas F.R., Fukushima J.T., Park C.H. (2016). Effect of perioperative goal-directed hemodynamic resuscitation therapy on outcomes following cardiac surgery: a randomized clinical trial and systematic review. Crit Care Med.

[28] Li P., Qu L.P., Qi D., Shen B., Wang Y.M., Xu J.R. (2017). Significance of perioperative goal-directed hemodynamic approach in preventing postoperative complications in patients after cardiac surgery: a meta-analysis and systematic review. Ann Med.

[29] Meersch M., Schmidt C., Hoffmeier A., Van Aken H., Wempe C., Gerss J. (2017). Prevention of cardiac surgery-associated AKI by implementing the KDIGO guidelines in high risk patients identified by biomarkers: the PrevAKI randomized controlled trial. Intensive Care Med.

[30] Alshaikh H.N., Katz N.M., Gani F., Nagarajan N., Canner J.K., Kacker S. (2018). Financial impact of acute kidney injury after cardiac operations in the United States. Ann Thorac Surg.

[31] Dushianthan A., Knight M., Russell P., Grocott M.P. (2020). Goal-directed haemodynamic therapy (GDHT) in surgical patients: systematic review and meta-analysis of the impact of GDHT on post-operative pulmonary complications. Perioper Med (Lond).

[32] García M.I., Romero M.G., Cano A.G., Aya H.D., Rhodes A., Grounds R.M. (2014). Dynamic arterial elastance as a predictor of arterial pressure response to fluid administration: a validation study. Crit Care.

[33] Hobson C.E., Yavas S., Segal M.S., Schold J.D., Tribble C.G., Layon A.J. (2009). Acute kidney injury is associated with increased long-term mortality after cardiothoracic surgery. Circulation.

[34] Brown J.R., Parikh C.R., Ross C.S., Kramer R.S., Magnus P.C., Chaisson K. (2014). Impact of perioperative acute kidney injury as a severity index for thirty-day readmission after cardiac surgery. Ann Thorac Surg.

[35] Vives M., Hernandez A., Parramon F., Estanyol N., Pardina B., Muñoz A. (2019). Acute kidney injury after cardiac surgery: prevalence, impact and management challenges. Int J Nephrol Renovasc Dis.

[36] Katz N.M., Kellum J.A., Ronco C. (2019). Acute kidney stress and prevention of acute kidney injury. Crit Care Med.

[37] Cummings J.J., Shaw A.D., Shi J., Lopez M.G., O'Neal J.B., Billings F.T. (2019). Intraoperative prediction of cardiac surgery-associated acute kidney injury using urinary biomarkers of cell cycle arrest. J Thorac Cardiovasc Surg.

[38] Engelman D.T., Crisafi C., Germain M., Greco B., Nathanson B.H., Engelman R.M. (2020). Using urinary biomarkers to reduce acute kidney injury following cardiac surgery. J Thorac Cardiovasc Surg.

